# An embodied cognition based model of medical experts’ tacit knowledge: structure, hierarchies, and transformation

**DOI:** 10.3389/fdgth.2026.1779044

**Published:** 2026-04-22

**Authors:** Hailing Zhou, Xinyue Chang, Xiaoyang Zhou, Jin Shi, Zuojian Zhou, Sheng Zhong

**Affiliations:** 1 School of Information Management, Nanjing University, Nanjing, China; 2 Department of Blockchain R&D, China Mobile Zijin (Jiangsu) Innovation Research Institute, Nanjing, China; 3 School of Artificial Intelligence and Information Technology, Nanjing University of Chinese Medicine, Nanjing, China; 4 Jiangsu Province Engineering Research Center of TCM Intelligence Health Service, Nanjing University of Chinese Medicine, Nanjing, China; 5 National Key Lab for Novel Software Technology, Nanjing University, Nanjing, China

**Keywords:** contextual, embodied cognition, medical expert, tacit knowledge, transformation mechanism

## Abstract

**Background:**

Tacit knowledge plays a crucial role in clinical decision-making and medical innovation, particularly through experience-based and practice-oriented expertise. However, existing research has not yet provided a sufficiently integrated framework to explain how such knowledge is structured and transformed within medical practice.

**Methods:**

Grounded in embodied cognition theory, this study constructs a medical experts' tacit knowledge model encompassing four key elements of expert agent, context, thinking, and action. Building upon the layered perspective of the onion model, the study organizes tacit knowledge across three levels and explains its dynamic and bidirectional transformation.

**Results:**

The resulting framework integrates embodied experience, cognitive processes, and clinical practice into a coherent system. A case analysis of the acupuncture expert Wang Leting and his “Lao Shi Zhen” prescription is used to illustrate how the model operates in practice.

**Conclusion:**

The study provides a systematic perspective for understanding medical experts' tacit knowledge and offers theoretical insights for medical education, knowledge transmission, and clinical decision support.

## 1 Introduction

Medical experts play a pivotal role in clinical practice, research, and education. They are not only the direct providers of healthcare services but also the principal agents in the transmission, preservation, and innovation of medical knowledge. Medical expertise consists of both explicit and tacit knowledge. Explicit knowledge can be articulated through textbooks, clinical guidelines, and formal education, whereas tacit knowledge is grounded in experience, intuition, and clinical practice.

According to knowledge management theory, tacit knowledge constitutes the major component of human knowledge (1). It is rooted in individual experience and interaction, making it difficult to codify or transmit through formal instruction. Empirical studies across fields such as healthcare, architecture, business management, and education have shown that tacit knowledge underpins effective professional action and expert performance (2). Polanyi's notion of personal knowledge that “we know more than we can tell” (3) further highlights its implicit and experiential nature. In medical domain, this form of knowing enables experts to make rapid and adaptive decisions in situations characterized by ambiguity, uncertainty, and risk (4).

The rapid development of artificial intelligence (AI), particularly deep learning and large language models, has profoundly advanced diagnostic assistance, medical imaging, and drug discovery. However, AI systems primarily rely on explicit, codified knowledge and large-scale structured data, which limits their ability to fully capture the complexity of clinical decision-making, including situational variability and physician—patient interaction (5). In response to these limitations, recent advances in digital health have increasingly emphasized the integration of artificial intelligence with human clinical expertise. Contemporary research on explainable AI (XAI) highlights the importance of transparency and interpretability in clinical decision support systems, particularly for enhancing clinician trust and usability in high-stakes environments (6). At the same time, studies on clinical cognition indicate that effective medical decision-making depends on experiential knowledge and adaptive reasoning processes that are difficult to fully represent in data-driven models (7, 8). Furthermore, emerging work on human—AI collaboration underscores the need to combine algorithmic predictions with expert judgment to enhance diagnostic performance and mitigate cognitive bias (9, 10). These developments highlight that tacit knowledge constitutes a critical dimension for advancing human—AI collaborative intelligence in medicine.

Within healthcare research, several studies have explored tacit knowledge from organizational or disciplinary perspectives. Nursing research has examined how tacit knowledge shapes clinical intuition and judgment (11), while public health studies have analyzed its role in evidence-based decision-making (4, 12). In addition, Abidi et al. (13) developed the scenario-based TKAI system to capture and formalize clinicians' tacit insights. While these studies provide valuable contributions, they primarily focus on knowledge sharing, transformation, or computational extraction (60), with less attention to the underlying cognitive structures that generate and sustain expert knowledge. Recent theoretical efforts, such as Eraut's analyses of tacit and non-formal learning (14–16), emphasize the role of experience and reflection in expert performance. These perspectives suggest that tacit knowledge is not merely a by-product of skill accumulation, but a dynamic cognitive system that evolves through interaction between body, environment, and social context. However, systematic inquiry into its structural composition and transformation mechanisms in medical expertise remains limited.

Tacit knowledge is not only an epistemological construct but also a critical factor in diagnostic accuracy, adaptive expertise, and clinical innovation. Understanding how such knowledge is structured and transformed is important for improving medical education and expert knowledge management, and may also provide insights for future research on the human—AI interface in healthcare. To further advance this line of research, this study adopts a conceptual modeling approach, integrating embodied cognition theory with insights from expert practice to develop an interpretive framework for analyzing tacit knowledge structure and transformation in healthcare domain.

This study makes three main contributions. First, grounded in embodied cognition theory, it proposes a conceptual model of medical experts' tacit knowledge, comprising expert agent, context, thinking, and action. The model integrates subjective, situational, cognitive, and behavioral aspects of expertise into a unified framework, rather than treating them as separate elements. Second, building on the layered perspective of the onion model, this study adopts a three-layer structure to clarify the internal organization of tacit knowledge. By integrating this layered structure with the four-dimensional framework, the model provides an operational representation of knowledge transformation pathways and offers a systematic approach for analyzing expert knowledge in clinical practice.Third, the study explicates the bidirectional transformation mechanisms across the three layers, conceptualizing knowledge evolution as a continuous and interactive process. This perspective contributes to a more systematic understanding of how tacit knowledge develops and transforms, and provides a theoretical basis for applications in medical education and knowledge management.

The remainder of this paper is organized as follows. Section 2 reviews the relevant theoretical foundations, including research on tacit knowledge and embodied cognition. Section 3 presents the proposed model, elaborates its operational mechanisms, and demonstrates its applicability through a case-based analysis. Section 4 concludes the paper by summarizing the main findings and discussing the theoretical implications and potential directions for future research.

## 2 Theoretical foundations

### 2.1 Individual tacit knowledge

#### 2.1.1 Concept of individual tacit knowledge

The study of tacit knowledge originates from philosophical inquiry and has developed along three main trajectories, the Polanyian, Wittgensteinian, and phenomenological-hermeneutic traditions (17). From a Polanyian perspective, tacit knowledge represents a form of experiential knowing, a personal understanding that precedes formal articulation. Polanyi's classic metaphor of learning to ride a bicycle illustrates the distinction between knowing how and knowing that. The former relies on practical coordination and intuitive adjustment, while the latter refers to propositional knowledge that can be explicitly stated (3, 18). In this view, knowledge is grounded in personal participation and practical experience.

The Wittgensteinian school further refines this view by distinguishing two forms of tacit knowledge: strong and weak. Strong tacit knowledge is inherently inexpressible, while weak tacit knowledge can be explicitly articulated but is typically left unspoken in practice (19). Meanwhile, the phenomenological-hermeneutic tradition traces tacit knowing to pre-reflective human experience. Husserl identified its roots in unconscious cognition, and Heidegger emphasized that understanding arises through human engagement with the world (17). Together, these perspectives suggest that tacit knowledge is not simply hidden information but a mode of being and acting. Building on these philosophical foundations, later scholars such as Nonaka extended the concept to applied and organizational contexts. Nonaka characterized tacit knowledge as highly personal, difficult to formalize or transfer, yet essential to creativity and innovation (1).

Across the literature, tacit knowledge exhibits several defining features. It is rooted in personal experience, difficult to codify, crucial for expert judgment and innovation, and integrates cognitive, emotional, and skill-based components (20–23).

#### 2.1.2 The structure of tacit knowledge

Building upon its conceptual foundations, scholars have increasingly examined the internal composition of tacit knowledge to develop systematic models that describe its components and relationships. Existing frameworks summarized in Table 1 generally adopt binary, triadic, or multidimensional structures, emphasizing aspects such as skills, experience, intuition, cognition, and values.

**Table 1:** Representative structural models of tacit knowledge.

Model type	Dimensions of Tacit knowledge	Applicable population	Reference
Triadic model	Knower, Subsidiary Awareness, Focal Awareness	General Individuals	(3)
Three-dimensional model	Content Dimension: managing the self, managing others, managing tasks;@Context Dimension: local context, holistic context;@Orientation Dimension: idealism, pragmatism	General Individuals	(24)
Binary model	Cognitive Dimension and Technical Dimension	General Individuals	(1)
Five-dimensional model	Metacognitive, value, emotional, interpersonal, and skill dimensions	General Individuals	(25)
Four-tier six-dimensional model	Metacognitive, value, operational skill, emotional, interpersonal, and sociocultural dimensions	Civil servants	(26)
Six-dimensional model	Learning strategies, clinical skills, interpersonal interaction, emotional–affective aspects, value orientation, and TCM cultural context	TCM professionals	(59)

From a knowledge management perspective, Nonaka divided tacit knowledge into technical and cognitive dimensions, highlighting the experiential and mental components that shape individual expertise (1). From an epistemological standpoint, Polanyi proposed a triadic structure consisting of focal awareness, subsidiary awareness, and the knower, emphasizing the perceptual and integrative processes involved in tacit knowing (3). Expanding this perspective through empirical research in organization, Wagner and Sternberg developed a three-dimensional model integrating content, context, and orientation to explain how tacit knowledge supports practical problem solving (24).

Subsequent studies further refined these frameworks within specific discipline. For instance, Li (25), Wang (26), and Zeng (59) proposed multidimensional models that incorporate elements such as metacognition, emotion, values, interpersonal interaction, and sociocultural factors, reflecting the complexity of tacit knowledge in professional practice. In the field of Traditional Chinese Medicine (TCM), Zeng's six-dimensional model highlights the integration of clinical experience with cultural cognition, illustrating how domain-specific knowledge systems shape tacit understanding.

To move beyond the static and classificatory nature of earlier models, Dan Asher and Micha Popper proposed a more dynamic and hierarchical “onion model” of tacit knowledge (27). This model conceptualizes tacit knowledge as comprising three interactive layers, which are unconscious, reflective, and conscious, illustrating a progression from deeply ineffable to partially articulable forms. By introducing hierarchical differentiation, the onion model provides a useful perspective for understanding the transformation of tacit knowledge. However, it primarily focuses on layer distinctions, offering limited analysis of the internal structural elements and their interactions in the development of expert knowledge.

In summary, prior models have advanced the understanding of individual tacit knowledge by introducing diverse structural dimensions. The onion model, in particular, contributes a hierarchical perspective on tacit knowledge transformation. However, existing frameworks remain largely descriptive and emphasize structural categorization, with insufficient attention to how different elements interact during knowledge development and application. In addition, the integration of multiple dimensions into a unified analytical framework remains underexplored, particularly in the context of medical expertise.

### 2.2 Embodied cognition theory

Embodied cognition departs from the traditional representational-computational view of the mind (28). It holds that cognition is not confined to brain processing but emerges from the dynamic interaction of perception, bodily action, and environmental context (29). Within this paradigm, embodiment, situatedness, and enaction serve as the three core concepts that provide complementary perspectives on how cognition is constituted.

Embodiment refers to the idea that cognitive processes are grounded in the body's sensorimotor capacities. Rather than being purely abstract or symbolic, cognition is shaped by perception and action, with the body functioning as a constitutive component of cognitive activity. Through continuous interaction with the environment, individuals develop and refine perceptual and motor skills that directly influence how information is processed and understood (30, 31).

Situatedness emphasizes that cognition always occurs within specific physical, social, and cultural settings. Cognitive activity is not context-free but is embedded in real-world situations where individuals draw upon available environmental and social resources to guide action (32). From this perspective, cognition is inseparable from the conditions under which it takes place, and understanding cognitive processes requires attention to these situational factors.

Enaction highlights the dynamic and emergent nature of cognition. It posits that cognition arises through continuous interaction between an organism and its environment (57), rather than through linear information processing. Knowledge is actively generated through cycles of perception and action, in which individuals both respond to and reshape their environment (33). This process underscores the adaptive and self-organizing character of cognitive activity.

In summary, embodied cognition redefines the cognitive subject by emphasizing the integration of bodily processes and environmental interaction in cognitive activity. Rather than viewing cognition as purely internal information processing, this perspective highlights the role of perception—action coupling and real-world engagement in shaping how knowledge is formed and applied. As a result, embodied cognition has significantly influenced a wide range of disciplines, including cognitive science, neuroscience, artificial intelligence, and psychology (34), providing an important theoretical lens for examining cognitive processes in practice-oriented domains.

### 2.3 The relationship between embodied cognition and tacit knowledge

Recent scholarship has increasingly emphasized the experiential, and generative nature of tacit knowledge, highlighting its close association with bodily experience and real-world practice (35, 36). From the perspective of embodied cognition, the formation of tacit knowledge can be understood as a continuous process in which perception, emotion, and action are integrated during interaction with the environment. Through ongoing engagement in practice, individuals continually reinterpret and reorganize their implicit understanding. When relevant situational cues are activated, tacit knowledge can be rapidly mobilized through intuitive and experience-based responses (37).

Empirical studies from various domains have further illustrated how sensorimotor involvement contributes to the development of tacit understanding. For instance, Xu (38), studying somatosensory educational games, demonstrated that enhanced physical engagement can facilitate the transformation of operational experience into deeper tacit knowledge. These findings suggest that embodied cognition processes play an important role in the formation and evolution of tacit knowledge, particularly in practice-oriented environments.

From this perspective, the perception-action loop emphasized in embodied cognition theory corresponds closely with the dynamic development and application of tacit knowledge. Rather than being static or fully pre-formed, tacit knowledge gradually evolves as individuals refine their understanding through repeated practice and reflection. Considering tacit knowledge through the lens of embodied cognition therefore helps clarify the mechanisms through which it is generated and developed.

Building on the above discussion, this study integrates the hierarchical perspective of the onion model with key insights from embodied cognition theory to construct a structural model of medical experts' tacit knowledge. Different from earlier descriptive frameworks, the proposed model highlights the interactive and process-oriented characteristics of tacit knowledge. It not only retains the hierarchical distinction among unconscious, reflective, and conscious layers but also systematically incorporates expert agency, context, thinking, and action as core structural components. By clarifying both the structural components and their interactions, the model provides a clearer analytical framework for examining the organization and transformation of medical experts' tacit knowledge.

## 3 Construction of the tacit knowledge model for medical experts

In high-risk and uncertain decision-making contexts, society relies heavily on experts to provide guidance for complex and critical tasks. When standardized procedures and conventional methods fall short, experts can still make rapid and reliable judgments amid ambiguity, conflict, or incomplete information, serving as a key cognitive resource for addressing complex challenges (39). This capacity arises from experts' structured and flexible knowledge networks, which are organized into deeply interconnected cognitive systems (40) and developed through long-term professional experience and deliberate practice (41). Consequently, when confronted with ill-defined problems, experts can reorganize their cognitive pathways, reconstruct problem representations, and, through analogical or case-based reasoning, mobilize prior experiential resources to generate effective solutions (42).

In the medical domain, expert knowledge comprises both explicit theoretical knowledge and tacit knowledge accumulated through extensive clinical experience. For instance, in endovascular treatment of intracranial aneurysms, expertise relies not merely on procedural steps but on situational judgment in coil selection, catheter positioning, and intraoperative manipulation, which cannot be fully verbalized (43). Although tacit knowledge may resist formal codification and fall outside the strict framework of evidence-based medicine, its practical efficacy in clinical contexts has been widely recognized (13, 58).

Building on these considerations, this study proposes a hierarchical and interactive model of medical experts’ tacit knowledge. The model conceptualizes tacit knowledge as a hierarchical and interactive structure composed of three layers and four core elements. The context-based unconscious layer, the interaction-based reflective layer, and the perception-based conscious layer represent different levels of awareness and articulation in expert cognition. Across these layers, tacit knowledge is constituted by four interrelated elements of expert agent, context, thinking, and action, which together describe how experts perceive situations, process information, and generate clinical responses through embodied interaction.

### 3.1 Tacit knowledge structure of medical experts

**Figure 1:**
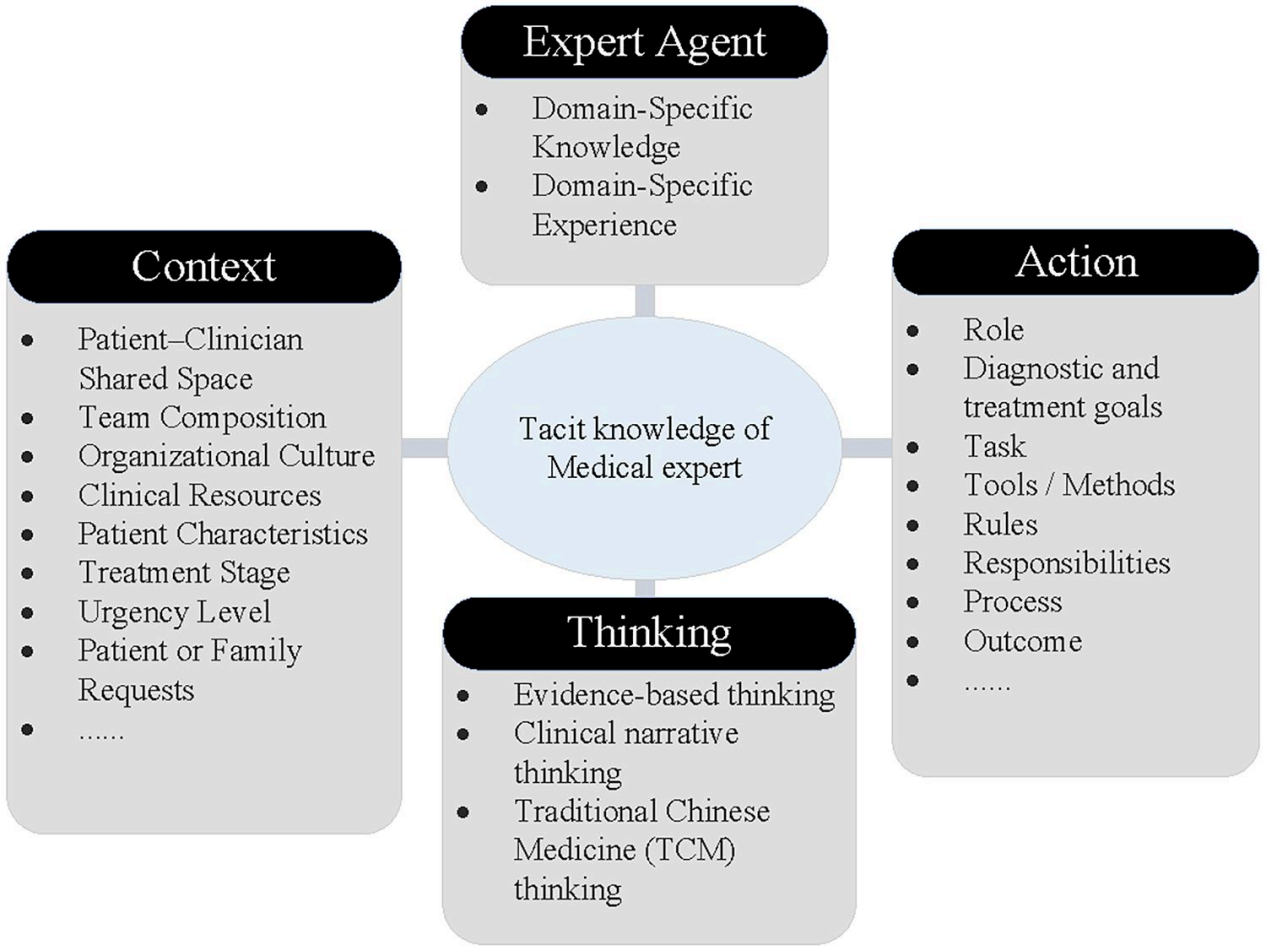
Tacit knowledge structure of medical expert.

As discussed in the theoretical foundations above, tacit knowledge represents the integrated manifestation of body, perception, and situational interaction. As illustrated in Figure 1, this study proposes a tacit knowledge structure for medical experts from the perspective of embodied cognition. The structure consists of four interrelated and nested elements, which are expert agent, context, thinking, and action.

#### 3.1.1 Expert agent element

Experts are widely recognized as possessing highly structured knowledge systems, exceptional pattern-recognition abilities, flexible case-based reasoning skills, and strong metacognitive awareness, enabling them to organize and apply knowledge efficiently in complex clinical situations (44). In this study, the expert agent element refers to the individual medical expert as the central source and carrier of tacit knowledge. Two main characteristics define this element. First, experts possess a solid foundation of explicit professional knowledge, including theoretical foundations, disease mechanisms, diagnostic criteria, and procedural standards, which provide a robust cognitive framework for clinical reasoning. Second, through extensive clinical practice, experts accumulate experiential resources. They further form implicit, uncodified cognitive strategies, interpretive tendencies, and operational habits that serve as the core of their tacit knowledge system.

The expert agent thus functions not only as the generator of tacit knowledge but also as its primary mediator in clinical practice. On the one hand, through repeated engagement with complex medical cases, experts gradually develop practical heuristics that guide diagnosis and treatment. On the other, these heuristics are typically conveyed through demonstration, mentoring, and case discussion, rather than through formal instruction, forming an important pathway for knowledge transfer within medical teams (45).

#### 3.1.2 Context element

From the perspective of embodied cognition, tacit knowledge is closely linked to the situations in which professional activities occur. Recent research further suggests that context does not merely influence knowledge activation but constitutes a structural component of the tacit knowledge system itself. Cognitive science indicates that when external environments and internal mental states form a functional coupling that co-drives cognition, context becomes an integral part of the cognitive system (46). Empirical research in neuroscience has shown that embodied simulation serves as the basis for understanding others' behaviors (47), while studies of complex work environments indicate that well-designed situations often contain distributed knowledge resources. Moreover, experts' performance deteriorates drastically when removed from their typical working contexts, demonstrating that context is indispensable to their competence systems.

For example, Ladds et al. (48) conducted in-depth interviews with community healthcare practitioners and found that clinical knowledge generation depends heavily on patient encounters and workplace contexts. They also noted that emerging contexts such as telemedicine significantly reshape clinicians' judgment patterns and experiential learning. Similarly, Haregu et al. (49) emphasized that context-embedded practical wisdom is essential for sustainable knowledge renewal in complex healthcare interventions.

Therefore, in this study, context is treated not simply as an external condition but as an important element of medical experts' tacit knowledge. It includes observable cues such as gestures, gaze, emotional tone, and response timing (50), as well as broader clinical environment, patient characteristics, stages of diagnosis and treatment, resource accessibility, team configuration, organizational culture, time pressure, and patient expectations. By shaping the conditions for knowledge generation, transmission, and application, context endows tacit knowledge with flexibility, adaptability, and uniqueness. These qualities explain why tacit knowledge is difficult to codify and formalize.

#### 3.1.3 Thinking element

Medical thinking refers to the cognitive processes through which medical experts interpret clinical information and formulate diagnostic or therapeutic strategies. It involves synthesizing professional knowledge, patient conditions, contextual variables, and clinical experience to make diagnostic and therapeutic decisions. Two major types of medical thinking commonly observed in clinical practice are Traditional Chinese medicine (TCM) thinking and modern medical thinking.

TCM thinking is characterized by analogical and holistic reasoning. It interprets external manifestations such as symptoms, tongue appearance, and pulse conditions as indicators of internal physiological states. progressing through four stages: observation, representation, analogy, and abstraction. This reasoning typically progresses through a process of observation, interpretation, analogy, and abstraction, emphasizing pattern recognition and accumulated clinical experience. Modern medical thinking primarily includes evidence-based and narrative modes of reasoning. Evidence-based thinking relies on statistical inference, standardized guidelines, and technical precision, emphasizing objectivity and reproducibility. In contrast, narrative thinking focuses on patients' lived experiences and clinical interpretation through dialogue, empathy, and contextual understanding (51).

Recent developments in precision medicine increasingly emphasize the integration of these reasoning approaches, combining the analytical rigor of evidence-based medicine with the interpretive perspective of narrative reasoning (52). In clinical practice, evidence-based reasoning supports scientific decision-making, while narrative reasoning helps clinicians better understand patient conditions and improve communication (53).

#### 3.1.4 Action element

From the lens of embodied cognition, knowledge is not limited to abstract representations or linguistic symbols but is enacted through bodily and operational practices. Medical experts' tacit knowledge is often activated through performance, where perception, intuition, and situational feedback converge during clinical operations. Preferences in tool handling, coordination rhythms, and sensitivity to subtle clinical cues exemplify this embodied activation of tacit knowledge.

The action element thus captures both the manifestation and the operational logic of tacit knowledge, encompassing how experts generate, validate, and adapt their understanding through practice. Drawing on Activity Theory (54), this element can be analyzed across eight categories of role, clinical goals, tasks, tools/methods, rules, responsibilities, process, and outcomes. For example, in emergency care, experts must rapidly define therapeutic objectives, select appropriate tools, coordinate task distribution, and flexibly adapt formal rules through embodied judgment. Under time pressure, the ability to prioritize, interpret non-verbal cues, and respond intuitively to unexpected events is critical for effective decision-making.

#### 3.1.5 Summary of the tacit knowledge system

In summary, the tacit knowledge system of medical experts comprises four nested elements. The expert agent acts as the knowledge holder and generator. The context provides the dynamic environment in which knowledge is situated and enacted. The thinking integrates scientific rationality with humanistic intuition. Action embodies and transmits knowledge through practice. Together, these elements constitute a tightly integrated cognitive system, forming the structural foundation of medical experts’ tacit knowledge.

### 3.2 Mechanism of tacit knowledge transformation in medical experts

**Figure 2:**
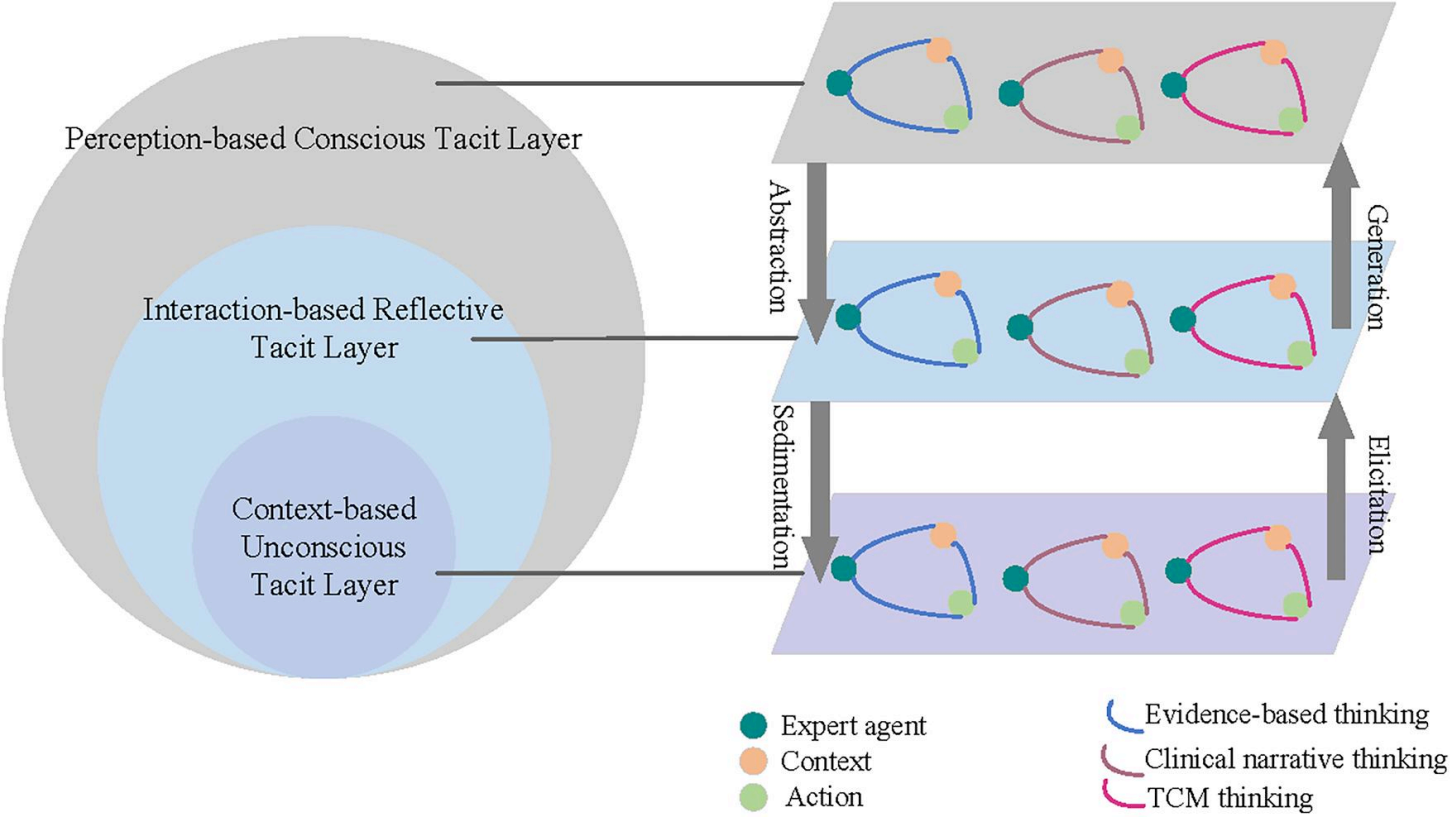
Tacit knowledge transformation mechanism.

Tacit knowledge is often activated through engagement with patients, teams, and iterative practice. Drawing on the concept of body-perception—action coupling from embodied cognition theory, this study proposes a mechanism of tacit knowledge transformation. As illustrated in Figure 2, the mechanism organizes tacit knowledge into three hierarchical layers: (1) context-based Unconscious Tacit Layer; (2) interaction-based Reflective Tacit Layer; (3) perception-based Conscious Tacit Layer. These layers form a progression from the implicit and experience-driven knowledge to reflective, partially articulable expertise. Arrows indicate bidirectional transitions between layers through internalization and externalization processes. Through clinical practice, reflection, and feedback, tacit knowledge is continuously refined, with some elements potentially transitioning into explicit, teachable knowledge. the transformation mechanism proceeds through three stages.

#### 3.2.1 Hierarchical structure of tacit knowledge

The transformation among three layers forms a continuous cycle, through which the overall structure of tacit knowledge can be more precisely delineated. Table 2 presents an illustration of the hierarchical structure of medical experts' tacit knowledge.

**Table 2:** Hierarchical description of medical experts’ tacit knowledge.

Layer	Characteristics	Representative content
Unconscious tacit layer	Rooted in embodied practice; highly intuitive; difficult to verbalize or formalize.	Diagnostic intuition, procedural skill, crisis response, perceptual acuity, tacit teamwork coordination.
Reflective tacit layer	Formed through reflective synthesis of experience; involves deeper personal understanding.	Complex decision strategies, procedural optimization, error reflection, adaptation to new techniques, research inspiration.
Conscious tacit layer	Semi-explicit knowledge; partially articulable but dependent on experiential grounding.	Semi-explicit diagnostic cues, cultural adaptation strategies, non-standard case reasoning, patient communication patterns, efficacy evaluation methods.

##### 3.2.1.1 Context-based unconscious tacit layer

The unconscious layer represents the innermost core of tacit knowledge, encompassing knowledge that is intuitive, automatic, and difficult to verbalize. It develops through extensive clinical exposure, forming a repository of operational sequences, decision heuristics, and anticipatory judgments (55). In medical contexts, experts' mastery of operational sequences, decision points, and crisis responses rarely derives from deliberate reasoning; rather, they know how to act without necessarily knowing why. This competence is grounded in fine-grained, embodied sensitivity acquired through long-term interaction with patients, instruments, and teams, representing a form of “knowledge-in-action” (56). Typical manifestations include: intuitive recognition of disease patterns, seamless procedural coordination, inference from subtle patient cues, and rapid adaptation in high-pressure situations. Knowledge at this level is enacted rather than formally articulated.

##### 3.2.1.2 Interaction-based reflective tacit layer

The reflective tacit layer occupies the intermediate level of the tacit knowledge hierarchy, serving as a bridge between unconscious behavioral responses and conscious experiential articulation. Reflection, in essence, is a process of cognitive elaboration upon experience, which is recalling, selecting, linking, and internalizing fragments of past practice to construct deeper understanding and meaning. This layer originates primarily from the action-reflection-reaction cycle (56), where continuous interaction is the core mechanism. Through ongoing communication with patients, collaborative feedback within teams, and evaluation of clinical outcomes, experts integrate and reconstruct their experiences, forming strategic knowledge that guides future practice.

Unlike the unconscious layer, knowledge in this domain involves partial conscious awareness, though it remains unsystematic and informal. It often manifests as heuristic rules, judgment patterns, or case-based reasoning paths, strategic insights distilled from repeated clinical encounters. Examples include: balancing treatment efficacy and patient preferences, refining strategies from prior errors, adapting to new techniques, and identifying unresolved clinical problems.

##### 3.2.1.3 Perception-based conscious tacit layer

The conscious tacit layer, situated at the outermost level of the tacit knowledge structure, is characterized by awareness and partial articulability. Although experts can consciously recognize and sometimes verbalize this knowledge, it is typically confined to personal practice and has not yet been formalized or codified within organizational or academic systems. Knowledge at this level often arises from bodily movements, sensory feedback, emotional responses, and repeated procedural experience, through which experts develop refined perceptual sensitivity and technical dexterity. While they can describe “how” a task is performed, the underlying integrative logic and intuitive judgment remain difficult to express precisely. As embodied cognition theory suggests, human perception is not solely a neural process but also a product of active bodily engagement with the environment; thus, any change in bodily structure or perceptual mode alters the very way knowledge is expressed and generated (29).

Typical examples of knowledge within this layer include: articulating diagnostic cues, adjusting strategies for diverse patient populations, documenting case-based reasoning, and sharing practice insights in training or mentor ship. The conscious layer represents both the culmination of tacit learning and the potential source for codifying explicit knowledge.

#### 3.2.2 Characteristics of tacit knowledge transformation

Within the proposed three-layer framework, knowledge flows bidirectionally among layers through iterative practice and reflection, forming a self-sustaining cognitive loop that is continuously activated, verified, and refined in real clinical contexts.

##### 3.2.2.1 Dynamic interaction

Knowledge acquisition ultimately aims to guide action. Since knowledge originates in practice, it must return to practice to realize its value. In this study, experts' tacit knowledge evolves through continuous interaction among the three layers. From the outer to inner layers, consciously accessible tacit knowledge is integrated through reflection into reusable heuristics and judgment patterns, forming reflective tacit knowledge. Through repeated application, these heuristics are gradually internalized and automatized, evolving into intuitive behavioral responses, forming unconscious tacit knowledge. Conversely, from the inner to outer layers, intuitive reactions can be reconstructed through reflection and deliberate intervention, progressively externalized into perceptible, expressible strategies that may evolve into conscious tacit or even explicit knowledge. This bidirectional evolution highlights that each layer's formation depends on the preceding one, and cognitive advancement occurs through iterative cycles of practice and reflective abstraction.

##### 3.2.2.2 Context dependence

In medical practice, knowledge and behavior are always situated within complex environmental, social, and cultural settings. Medical experts adapt diagnostic and therapeutic strategies based on patient history, lifestyles, treatment preferences, or cultural backgrounds. Even when addressing the same disease, clinical decisions may differ across situations, reflecting the adaptive nature of tacit knowledge. Similarly, implicit coordination within medical teams emerges through prolonged joint practice and bodily synchronization. These forms of situated expertise reveal that tacit knowledge is activated by specific contexts rather than universally applied.

##### 3.2.2.3 Knowledge generation

The tacit knowledge also has a generative capacity. Each new clinical experience, especially those involving uncertainty or boundary-challenging cases, stimulates restructuring and renewal of prior knowledge. Through ongoing cycles of perception, action, and reflection, experts develop new diagnostic standards, procedural innovations, and collaborative approaches, allowing tacit knowledge to continually evolve. In contexts of clinical innovation or interdisciplinary collaboration, experts often transcend established cognitive boundaries, generating novel forms of practical wisdom through iterative experimentation (49).

In conclusion, the three-layer structure of medical experts' tacit knowledge does not constitute a closed hierarchy but rather a multi-directional, context-driven, and practice-generated system. Each layer fulfills a distinct function within the expert's overall cognitive architecture, while their dynamic interrelations drive the continuous deepening and expansion of knowledge. This systemic understanding not only elucidates the internal logic of expert cognition but also provides a theoretical and methodological foundation for identifying, transmitting, and activating tacit knowledge in medical education and practice.

#### 3.2.3 Pathways of tacit knowledge transformation

To operationalize the dynamic transformation mechanism of medical experts' tacit knowledge, this study encodes the hierarchical structure of tacit knowledge into a formalized system. This allows for a detailed, step-by-step mathematical description of the transformation pathways.

In this study, each level is defined as a knowledge state, represented by a vector K = {E, C, T, A}, composed of expert agent (E), context (C), thought (T), and action (A). The tacit knowledge of medical experts exists across three layers: the context-based unconscious tacit layer (K_1_), the interaction-based reflective tacit layer (K_2_), and the body-perception-based conscious tacit layer (K_3_). Two major transformation pathways are identified as internalization and externalization. Each pathway is a sequence of state transitions, governed by specific functions that map the elements {E, C, T, A} from one state to another.

##### 3.2.3.1 Internalization

This pathway describes the transition of knowledge from a conscious, deliberate state (K_3_) to an unconscious, automatic state (K_1_). The process in which deliberate thought compresses into intuition involves two distinct state transitions: Step 1 abstraction (K_3_ → K_2_). This step models the abstraction of specific experience into generalizable heuristics. Step 2 sedimentation (K_2_ → K_1_). This step models the embedding of these heuristics into the expert's unconscious intuition. The entire pathway can be represented as a composite function: K_1_ = Sedimentation[Abstraction(K_3_)], or in detail: K_3_{E_3_, C_3_, T_3_, A_3_} → K_2_{E_2_, C_2_, T_2_, A_2_} → K_1_{E_1_, C_1_, T_1_, A_1_}.

1. Step 1 Mathematical Description: The Abstraction Transition (K_3_ to K_2_)

This transition maps a concrete, situated experience into a reflective, socially-negotiated heuristic.

Input state: K_3_ = {E_3_, C_3_, T_3_, A_3_}, where

E_3_: The expert, acting with focused, conscious cognition.

C_3_: A specific, real-world clinical context.

T_3_: Deliberate, step-by-step analytical thought, e.g., differential diagnosis.

A_3_: Refined, deliberate actions, e.g., specific physical exam maneuvers.

Transition Function F_abstraction: This function is triggered by social interaction (C_2_). It takes the concrete A_3_ and T_3_ from the specific context C_3_ and abstracts them.

T_2_ = Abstract(T_3_, A_3_, C_2_): Through social reflection, the linear thought process T_3_ is condensed into a set of mental rules or heuristics T_2_. For example, the detailed logic of a differential diagnosis (T_3_) is abstracted into the heuristic “When symptom X and Y present together, always consider condition Z” (T_2_).

A_2_ = Generalize(A_3_, C_2_): The specific actions A_3_ are generalized into a class of actions. For instance, a specific surgical maneuver A_3_ becomes the general principle of “maintain tension on the tissue” (A_2_).

S_2_: The situation transforms from the physical patient C_3_ to a social/interactive context C_2_ (the discussion itself).

E_2_: The expert's knowledge base is now augmented with the newly formed heuristics T_2_.

Output state: K_2_ = {E_2_, C_2_, T_2_, A_2_}, where

E_2_: Expert with knowledge base enriched by new heuristics.

C_2_: The social/interactive context.

T_2_: Abstracted heuristics and decision rules.

A_2_: Generalized action principles.

(2) Step 2 Mathematical Description: The Sedimentation Transition (K_2_ to K_1_)

This transition maps reflective heuristics into unconscious intuitions, driven by repetition and validation.

Input state: K_2_ = {E_2_, C_2_, T_2_, A_2_}.

Transition Function F_sedimentation: This function models the effect of repeated, successful application of the heuristic T_2_ in real-world practice.

Let *n* be the number of successful applications of the heuristic T_2_ in new, concrete situations.

T_1_ = Condense(T_2_, *n*): As *n* increases, the conscious heuristic T_2_ condenses further. When *n* exceeds a threshold N_automatic, T_2_ transforms into T_1_, which is no longer a conscious rule but an instantaneous, unconscious pattern recognition.

A_1_ = Automatize(A_2_, *n*): Similarly, the generalized action principle A_2_ becomes an automatic motor response A_1_. The action shifts from being “thought about” to being “felt.”

C_1_ = Internalize(C_3_): The specific context C_3_ that originally triggered the heuristic is internalized as a contextual expectation C_1_. The expert no longer needs to see the situation as a set of abstracted features; they intuitively feel it.

E_1_: The heuristic is now sedimented into the subject knowledge base, becoming part of the expert's unconscious intuition E_1_.

Output state: K_1_ = {E_1_, C_1_, T_1_, A_1_},where

E_1_: The expert's enriched, unconscious knowledge base.

C_1_: Internalized contextual expectation.

T_1_: Instantaneous, intuitive pattern recognition.

A_1_: Automatic, embodied response.

##### 3.2.3.2 Externalization

This pathway describes the transition of knowledge from an unconscious intuition (K_1_) to a conscious, testable strategy (K_3_). It is a process of innovation, where a vague feeling is crystallized into a novel action. It involves two transitions: Step 1, elicitation (K_1_ → K_2_). A vague intuition is triggered and brought into conscious reflection. Step 2, crystallization (K_2_ → K_3_). The reflected idea is transformed into a concrete, exploratory action. The pathway is represented as: K_3_ = Crystallization[Elicitation(K_1_)], or K_1_{E_1_, C_1_, T_1_, A_1_} → K_2_{E_2_, C_2_, T_2_, A_2_} → K_3_{E_3_, C_3_, T_3_, A_3_}.

1. Step 1 Mathematical Description: The Elicitation Transition (K_1_ to K_2_)

This transition maps an unconscious alert into a conscious, reflective inquiry.

Input state: K_1_ = {E_1_, C_1_, T_1_, A_1_}, where

E_1_: The expert with a rich, intuitive knowledge base.

C_1_: An internalized expectation of context.

T_1_: Unconscious intuition.

A_1_: Automatic actions.

Triggering Condition: An anomaly from the environment C_env creates a mismatch with the internalized expectation C_1_. The automatic action A_1_ fails to resolve the anomaly. This mismatch generates an intuitive alert.

Transition Function F_elicitation: This function models the process of bringing the intuition to the surface.

T_2_ = Articulate(T_1_, mismatch): The unconscious signal T_1_ is articulated into a conscious, reflective thought T_2_. For example, “Something feels wrong” (T_1_) becomes “What if the patient has a rare complication Y?” (T_2_). This is a hypothesis formation.

C_2_: The expert engages in social interaction (C_2_) to test this hypothesis. This could be an internal dialogue, a literature search, or a discussion with a colleague.

A_2_: The automatic response A_1_ is inhibited. The reflective action A_2_ becomes observation, information gathering, and mental simulation.

E_2_: The expert's knowledge base is now actively searching for links to the new hypothesis T_2_.

Output state: K_2_ = {E_2_, C_2_, T_2_, A_2_}, where

E_2_: Knowledge base in an active, searching state.

C_2_: The reflective context, internal or social.

T_2_: A conscious, articulated hypothesis or question.

A_2_: Reflective actions of observation or inquiry.

(2) Step 2 Mathematical Description: The Crystallization Transition (K_2_ to K_3_)

This transition maps a reflective hypothesis into a concrete, exploratory action in the real world.

Input state: K_2_ = {E_2_, C_2_, T_2_, A_2_}.

Transition Function F_crystallization: This function models the design and execution of a test for the hypothesis T_2_.

A_3_ = Operationalize(T_2_, C_2_): The reflective thought T_2_ is operationalized into a concrete, exploratory action A_3_. This action is designed to reshape the situation to test the hypothesis.

C_3_: The situation transitions from the reflective context C_2_ back to the real or simulated clinical context C_3_, which is now being acted upon by A_3_.

T_3_: The reflective thought T_2_ becomes the focused, conscious cognition T_3_ that guides the execution of A_3_ and interprets its results.

E_3_: The knowledge base E is reorganized based on the outcome of the action. If the hypothesis is confirmed, new cognitive links are formed. This generates a new problem-solving strategy.

Output state: K_3_ = {E_3_, C_3_, T_3_, A_3_}, where

E_3_: The expert's reorganized and expanded knowledge base.

C_3_: The new, reshaped situation.

T_3_: Focused, conscious cognition.

A_3_: Exploratory diagnostic or therapeutic actions.

##### 3.2.3.3 Generation of explicit knowledge

The transformation of tacit knowledge is defined by the dynamic circulation between these three knowledge states. Each complete pathway constitutes a learning cycle.

An Internalization cycle (K_3_ → K_2_ → K_1_) increases the system's “automaticity,” expanding the expert's intuitive base E_1_ through the function: E_1_(t + 1) = E_1_(*t*) ∪ {Sedimented_Heuristics}.

An Externalization cycle (K_1_ → K_2_ → K_3_) increases the system's “capacity for innovation,” revising the knowledge base with new strategies: E_3_(t + 1) = E_3_(t) ⊕ {New_Strategies}, where ⊕ denotes reorganization and expansion.

Through the continuous iteration of these dual pathways, experience is constantly sedimented into deeper intuition, while intuition is crystallized into innovative strategies. Over time, when a new strategy A_3_ proves robust across multiple cycles, it can be formalized. This is a final transition where the elements of K_3_ are codified into language, diagrams, or protocols. This codified knowledge is the explicit knowledge that enables dissemination and professional communication. This formalized cyclical mechanism, now with mathematically describable steps, drives the ongoing evolution and transcendence of individual cognitive competence.

### 3.3 Case study of the tacit knowledge model of medical experts

As an essential component of traditional Chinese medicine (TCM), acupuncture is deeply rooted in Chinese cultural traditions. Its distinctive meridian theory, holistic perspective, dialectical reasoning, and acupuncture techniques embody a vast body of highly individualized, experience-based, and tacit knowledge that is difficult to articulate, formalize, or standardize. Such knowledge is often accumulated through prolonged clinical practice, and gradually internalized into unique operational strategies and cognitive schemas through imitation, reflection, and repeated embodied experience. Consequently, it exhibits the typical characteristics of non-logicality, vagueness, subjectivity, and contextual dependence.

Professor Wang Leting, a renowned scholar at the Beijing College of Traditional Chinese Medicine (now Beijing University of Chinese Medicine), is one of the most distinguished figures in the history of Chinese acupuncture. Born into a family of traditional physicians, Wang studied under Chen Suqing and later pursued advanced medical training at the National Medical College of Beiping. Throughout his career, he dedicated himself to acupuncture practice and education, leaving behind numerous classical acupuncture prescriptions that have profoundly influenced the theoretical development and clinical practice of acupuncture in China.

**Figure 3:**
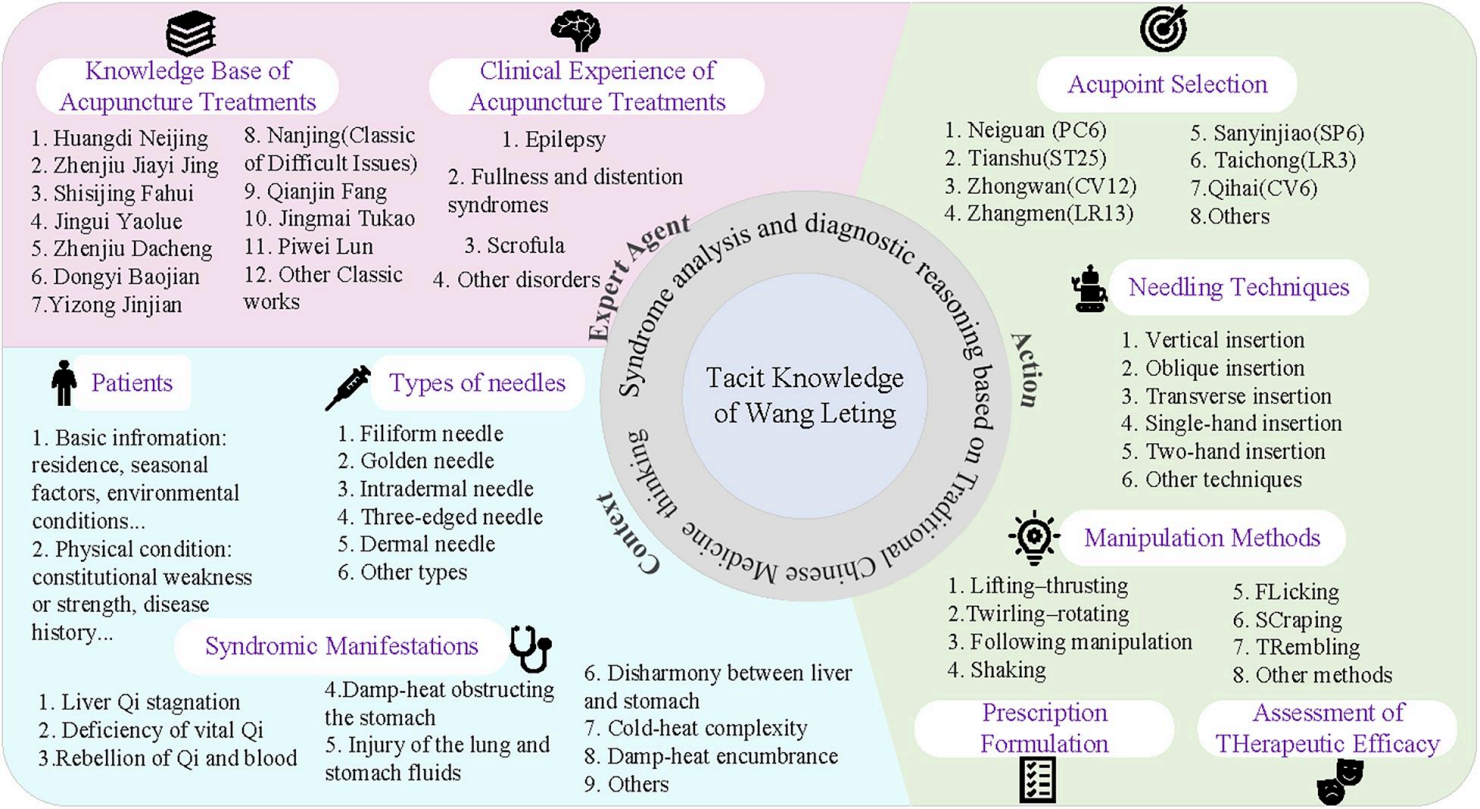
Tacit knowledge structure of Wang Leting.

This study takes Professor Wang Leting's tacit knowledge structure as a representative case. Figure 3 illustrates the constituent elements of his tacit knowledge unit, which correspond to the four elements of the model: expert agent, context, thinking, and action. First, the expert agent element is reflected in Wang's extensive professional knowledge and personal clinical experience. His mastery of classical medical texts such as Treatise on the Spleen and Stomach (Pi Wei Lun) and Prescriptions Worth a Thousand Gold Pieces (Qian Jin Fang), combined with decades of clinical observation, formed the foundational knowledge base that guided his diagnostic and therapeutic activities. Second, the context element is manifested in the specific clinical situations in which Wang practiced. Through long-term treatment of conditions such as scrofula, paralysis, and epigastric distension, he accumulated rich situational knowledge regarding disease patterns, patient characteristics, and treatment environments. These contextual factors continuously shaped and refined his therapeutic decisions. Third, the thinking element is represented by Wang's diagnostic reasoning and therapeutic strategies. His medical thinking integrated the symbolic cognition characteristic of Chinese medicine with the principles of syndrome differentiation and treatment, enabling him to interpret complex clinical manifestations and develop personalized treatment plans. Fourth, the action element is embodied in his acupuncture techniques and clinical operations. These include acupoint selection rules, needling angles, manipulation techniques, and specific point combinations. Such operational knowledge is largely tacit and cannot be fully conveyed through verbal explanation; rather, it must be acquired through observation, imitation, and repeated clinical practice.

**Figure 4:**
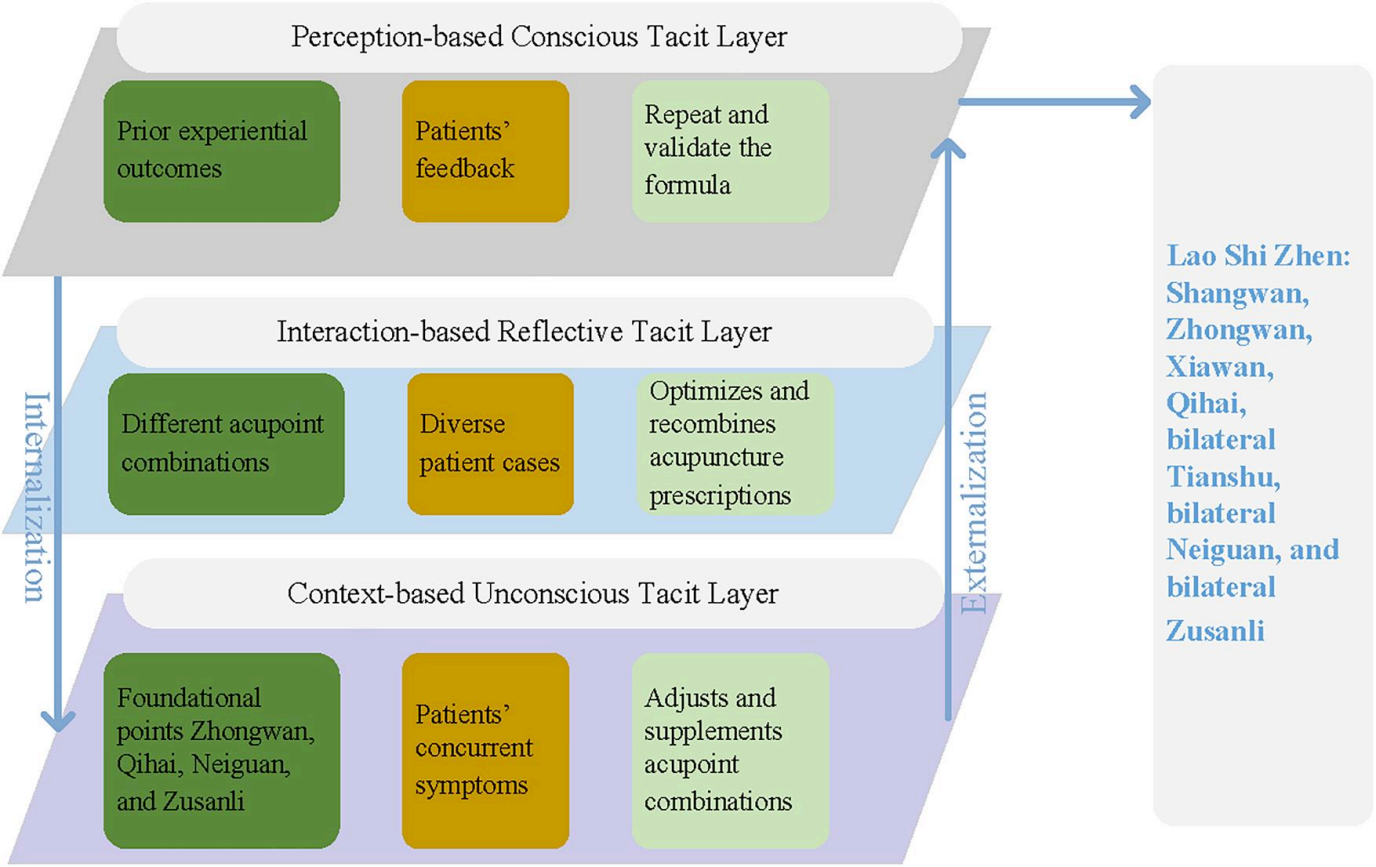
Formation pathway of the “Lao Shi Zhen” prescription.

Figure 4 further illustrates the formation process of Wang's well-known acupuncture prescription, the “Lao Shi Zhen,” which exemplifies the dynamic transformation process of tacit knowledge described in the proposed model. Through years of clinical experience, Wang observed that gastrointestinal diseases were highly prevalent and particularly responsive to acupuncture treatment. Initially, he selected traditional points such as Zhongwan, Qihai, Neiguan, and Zusanli, adjusting combinations based on patients' symptoms. At this stage, his knowledge primarily relied on conscious embodied practice (K_3_), in which clinical actions and sensory feedback guided decision-making. Through repeated treatment and accumulated experience, Wang gradually developed intuitive understandings of acupoint interactions and therapeutic effects. These intuitive insights represent the unconscious tacit layer (K_1_) formed through long-term clinical sedimentation. Meanwhile, through reflection on treatment outcomes and interaction with classical medical theories, Wang continually refined his diagnostic reasoning and therapeutic strategies. This process corresponds to the reflective tacit layer (K_2_), where experiential knowledge is reorganized and conceptualized through reflective thinking.

Through iterative cycles of action—reflection—revised action, Wang gradually optimized acupoint combinations, adding Shangwan, Xiawan, and Tianshu, thereby forming a more systematic acupuncture prescription. Eventually, drawing on the conceptual principles of Tonifying the Middle and Augmenting Qi Decoction (Bu Zhong Yi Qi Tang) and Regulating the Center Decoction (Tiao Zhong Yi Qi Tang) from Li Dongyuan's Treatise on the Spleen and Stomach, he translated herbal therapeutic principles into acupuncture equivalents. Around 1966, he finalized the “Lao Shi Zhen,” consisting of Shangwan, Zhongwan, Xiawan, Qihai, bilateral Tianshu, bilateral Neiguan, and bilateral Zusanli.

Overall, this case demonstrates how the four structural elements operate within the three-layer tacit knowledge system proposed in this study. The development of “Lao Shi Zhen” illustrates the bidirectional transformation between unconscious intuition, reflective reasoning, and conscious clinical practice, thereby providing empirical support for the hierarchical and dynamic characteristics of medical experts' tacit knowledge described in the proposed model.

## 4 Conclusion

Drawing on the theory of embodied cognition, this study proposes a model of medical experts' tacit knowledge structured around four elements and three hierarchical layers. The model systematically reveals the dynamic interactivity, contextual dependence, and continuous generativity inherent in the transformation of medical experts' tacit knowledge.

At the theoretical level, the model advances the understanding of tacit knowledge by elevating context and action from external influencing factors to constitutive elements equal in importance to the internal knowledge base of the subject. This ontological redefinition establishes the systemic and generative nature of tacit knowledge, providing a stronger philosophical foundation for comprehending its essence.

At the mechanistic level, the study delineates a bidirectional transformation pathway among the three layers of tacit knowledge, clarifying the key mediating processes that drive the internalization and externalization of expert cognition. In doing so, it conceptualizes the transformation of medical experts' tacit knowledge as an analyzable, intervenable, and practicable system.

However, the study acknowledges certain limitations in empirical validation. Future research will incorporate cross-disciplinary empirical analyses within diverse medical contexts, such as traditional Chinese medicine and Modern medicine, to further refine the manifestations and transformation conditions of tacit knowledge across different layers. Moreover, future work will explore the integration of artificial intelligence, multimodal data modeling, and knowledge representation technologies to achieve structured extraction, dynamic tracking, and assisted expression of tacit knowledge. Such efforts aim to advance the management of tacit knowledge toward greater intelligence, precision, and applicability.

## Data Availability

The original contributions presented in the study are included in the article/Supplementary Material, further inquiries can be directed to the corresponding authors.
